# A single nuclei atlas of aging human abdominal subcutaneous white adipose tissue

**DOI:** 10.21203/rs.3.rs-3097605/v1

**Published:** 2023-07-13

**Authors:** Lauren Sparks, Katie Whytock, Adeline Divoux, Yifei Sun, Maria Pino, Gongxin Yu, Steven Smith, Martin Walsh

**Affiliations:** Translational Research Institute, AdventHealth; Translational Research Institute, AdventHealth; Translational Research Institute, AdventHealth; Icahn School of Medicine at Mount Sinai; Translational Research Institute, AdventHealth; Translational Research Institute, AdventHealth; Translational Research Institute, AdventHealth; Icahn School of Medicine at Mount Sinai

## Abstract

White adipose tissue (WAT) is a robust energy storage and endocrine organ critical for maintaining metabolic health as we age. Our aim was to identify cell-specific transcriptional aberrations that occur in WAT with aging. We leveraged full-length snRNA-Seq to characterize the cellular landscape of human subcutaneous WAT in a prospective cohort of 10 Younger (≤ 30 years) and 10 Older individuals (≥ 65 years) balanced for sex and body mass index (BMI). We highlight that aging WAT is associated with adipocyte hypertrophy, increased proportions of resident macrophages (M2), an upregulated innate immune response and senescence profiles in specific adipocyte populations, highlighting CXCL14 as a biomarker of this process. We also identify novel markers of pre-adipocytes and track their expression levels through pre-adipocyte differentiation. We propose that aging WAT is associated with low-grade inflammation that is managed by a foundation of innate immunity to preserve the metabolic health of the WAT.

Aging is associated with a progressive decline in physiological function leading to augmented human pathology and vulnerability to death^1^. White adipose tissue (WAT) functions as a robust energy store, an endocrine organ that governs whole-body metabolic homeostasis and a tissue that regulates immune modulation and regeneration^2,3^. Impairments in WAT function lead to unfavorable WAT redistribution towards central abdominal stores^4^, ectopic lipid accumulation and subsequent peripheral insulin resistance in organs such as skeletal muscle and liver^5,6^, and low-grade chronic systemic inflammation^7,8^. Elevated cellular senescence^9–11^, reduced progenitor proliferation, impaired adipogenic potential of the progenitor pool^12^ and immune cell infiltration^13^ have all been purported as factors contributing to age-associated decline in WAT function. These prior analyses, however, have largely been restricted to *in vitro* assessments or targeted histological and FACS approaches. Single cell/nuclei and spatial transcriptomics in human subcutaneous WAT is a rapidly growing area of research^14–25^ with aging recently being explored in a limited scope^14^. Our aim was to advance these findings at single nuclei resolution by analyzing nuclei-specific transcriptional differences in WAT between Younger and Older adults. In this study, for the first time, we prospectively obtained abdominal subcutaneous WAT biopsies for snRNA-Seq analyses from individuals who were metabolically normal and were not undergoing surgery. We leveraged our recent advancements in full-length snRNA-Seq in human WAT which yields superior gene detection capabilities compared to prior single cell/nuclei RNA-Seq platforms and technologies such as 10X Genomics^24,26^, combined with histological and *in vitro* assessments, to delineate nuclei-specific aberrations in human WAT with aging.

## Results

**Figure 1:**
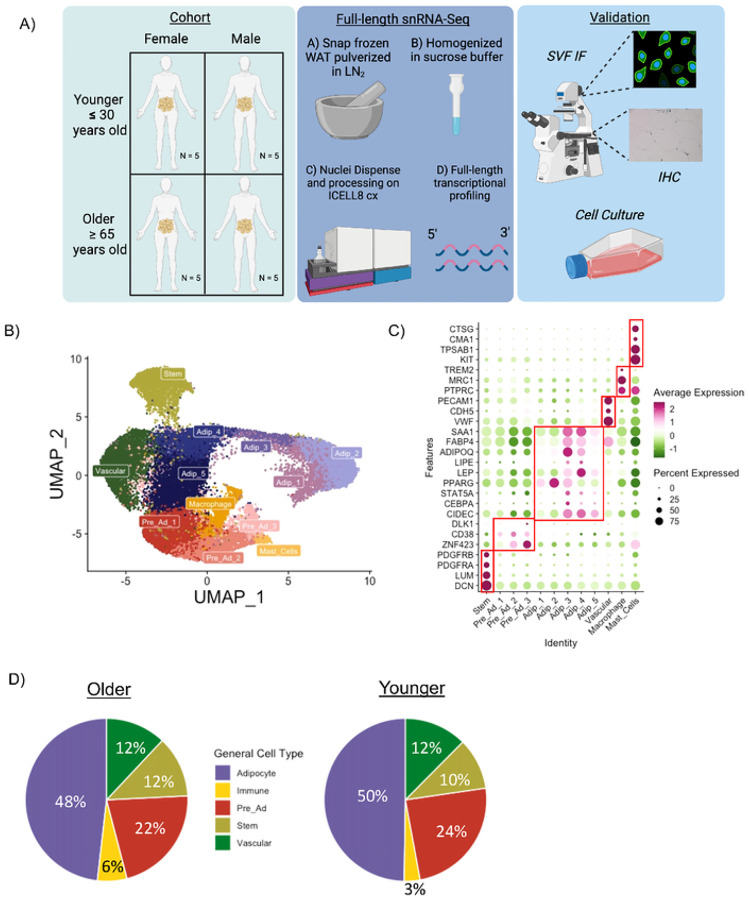
A single nuclei atlas of aging human abdominal subcutaneous white adipose tissue. Schematic overview of study design and methods (A). UMAP of 25,736 nuclei from abdominal subcutaneous white adipose tissue (WAT) of 10 Younger and 10 Older participants (B). Dotplot of known marker genes for each cell population (C). Cell composition differences in the main cell population between Older and Younger participants (D).

To explore the effects of aging on WAT, we performed full-length snRNA-Seq analysis on abdominal subcutaneous WAT from 10 Older (≥ 65 years old) and 10 Younger (≤ 30 years old) individuals balanced for sex and BMI (Figure 1A). All individuals were metabolically healthy ≤ (Table 1, **Supplementary Information 1**). WAT biopsies were performed at Translational Research Institute (TRI) for the sole purpose of interrogating cell-specific aberrations with aging (see methods for processing details). The Older group had greater levels of systolic blood pressure, waist circumference, waist-to-hip-ratio, plasma cholesterol, very-low-density lipoprotein, triglycerides, thyroid stimulating hormone and aspartate transaminase vs. the Younger group (P < 0.05); however, all values were in the normal range. There were no differences in BMI, plasma glucose, plasma free fatty acids (FFAs), serum insulin, serum CRP, or adipose tissue insulin sensitivity measured by ADIPO-IR^27,28^ between the Older and Younger groups, indicative of comparable metabolic health, particularly for the WAT.

### Aging human WAT single nuclei atlas

Our dataset included 25,736 nuclei composed of 12 different clusters (Figure 1B) with an average of 5304 genes detected per nuclei. Known cell markers were used for initial annotation (Figure 1C) revealing populations of stem cells, pre-adipocytes, adipocytes, vascular cells, macrophages and mast cells. By transcriptionally profiling the nuclei isolated from WAT, we determined cell composition of adipocytes and non-adipocyte cells using an array of markers that are not limited to pre-specific cell markers. The largest cell type proportion in WAT is adipocytes (~50%) followed by pre-ads (22–24%), vascular cells (~12%), stem cells (10–12%) and immune cells (3–6%) (Figure 1D). Each participant had every cell type quantified except for mast cells (**Extended Figure 1A-B**). The Older group in comparison to the Younger group had double the proportion of immune cells (Figure 1D) in agreement with previous findings^13^. There were no major cell compositional differences between males and females (**Extended Figure 1C**).

#### Unbiased annotation of cell types

**Figure 2:**
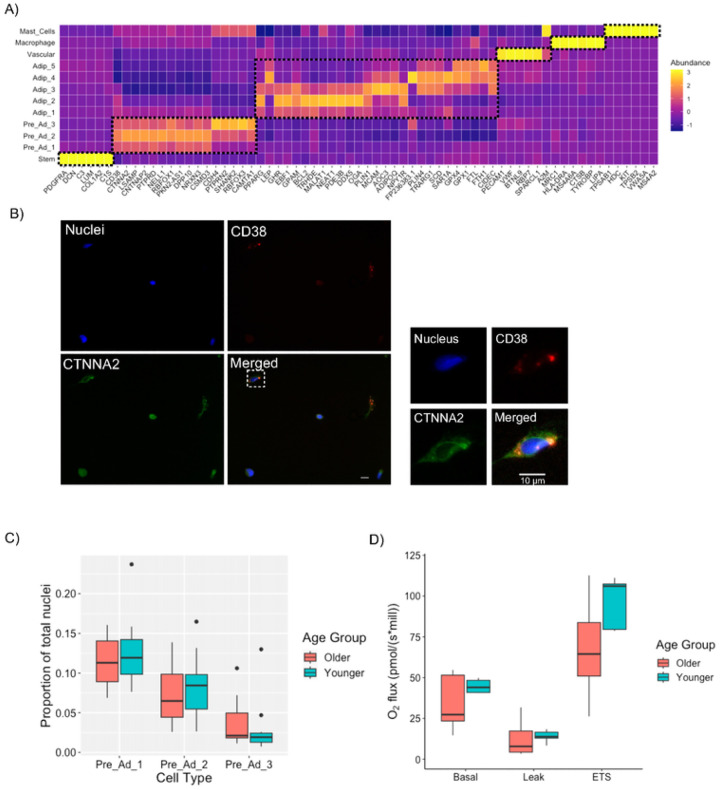
Full-length snRNA-seq reveals uncommon markers of pre-adipocytes. Heatmap of Top 5 upregulated genes in each cell population with known marker genes included as a reference (A). Immunofluorescence staining of SVF obtained from subcutaneous adipose tissue with putative marker CD38 and novel marker CTNNA2. Images obtained at 40x magnification (B). Cell composition of the different pre-adipocyte populations in relation to the total number of nuclei in the Older and Younger groups (C). Pre-Adipocyte respiratory capacity measured with high resolution respirometry from Younger (n = 5) and Older (n = 5) Male participants (D).

We performed an unbiased annotation of the cell clusters by running DGE analysis on each cluster to identify uncommon cell type markers. The top 5 DEGs for each cluster are highlighted in Figure 2A (Known cell markers of each cluster [i.e. *PDGFRA, CD38, LEP, PPARG, PECAM1, MRC1* and *TPSAB1*] are included as a reference for the main cell types. As anticipated, stem cells had the highest expression of *DCN, LUM* and *COL1A2*, vascular cells had the highest expression of *VWF*, macrophages had the highest expression of *HLA-DRA* and *LIPA*, and mast cells had the highest expression of *KIT*. Classical markers of adipocytes *ADIPOQ* and *PLIN1* were upregulated to varying degrees in all five adipocyte clusters. Classical pre-adipocyte markers such as *ZNF423, CD38* and *DLK1* were not in top DEGs for each pre-adipocyte population; but rather, uncommon markers such as *CTNNA2, RBFOX3, PTPRD* and *NRXN3* were highly expressed in our pre-adipocyte populations. Previous snRNA-Seq data sets in mouse and human WAT have identified pre-adipocyte populations within a subset of progenitor cells, usually marked by ICAM1 or CD36^20,23,29–32^; however, these previously identified pre-adipocytes still express known stem cells markers such as *PDGFRA, COL4A1, COL1A1*^20,29,31,32^ which should diminish upon commitment to the adipogenic lineage^33^. Therefore, these previously identified pre-adipocyte populations may not be committed pre-adipocytes. In contrast, our pre-adipocyte populations are transcriptionally distinct from our stem cell pool and have reduced expression of stem cell markers (*PDGFRA, LUM, DCN*; Figure 2A).

To validate our cluster annotations in the pre-adipocyte populations, we compared gene expression profiles to previously published literature. We recently published full-length scRNA-Seq on the stromal vascular fraction (SVF) of abdominal subcutaneous WAT and transcriptionally profiled the commitment of pre-adipocytes from pluripotent stem cells (Whytock *et al*., 2022). We integrated our current dataset (annotated as BA) with the scRNA-Seq SVF and ran pairwise correlation analysis of the gene expression profiles between the identified cell clusters. Gene expression profiles from our defined cell populations correlated strongly with each other from both data sets, including our pre-adipocyte populations (**black dashed box, Extended Figure 2A**). Recently, Emont *et al*.,^20^ published a single cell atlas of WAT from various mouse and human fat pads and depots, respectively. We filtered the Emont data set to only include human abdominal subcutaneous WAT and compared gene expression profiles of major cell clusters against our data set. Gene expression profiles from major cell populations highly correlated with each other between the two data sets (**Extended Figure 2B**). Interestingly, our pre-adipocyte populations correlated most strongly with the lymphatic endothelial cell (EC) cluster in the Emont data set (**black dashed box, Extended Figure 2B**). All three of our pre-adipocyte clusters did not express upregulated endothelial cell makers (*PECAM1, VWF, CDH5*) or the specific lymphatic EC marker *LYVE1*. We are therefore confident that our pre-adipocyte populations are not lymphatic ECs and are indeed pre-adipocytes based on expression of expected markers (*CD38, ZNF423 and DLK1*)^34–36^. Both Emont *et al*.^20^ and a recent meta-analysis of human scRNA-Seq data^25^ had more defined immune cell populations (NK/T/B cells) in comparison to our data set, which is likely driven by the manner in which we procure our abdominal subcutaneous WAT samples. Ours^24^ are the only human single cell/nuclei RNA-Seq data generated from subcutaneous WAT obtained via lipoaspiration of the superficial subcutaneous depot rather than during surgery, the latter of which likely induces tissue inflammation.

#### Pre-Adipocytes

The top five DEGs for each of the three pre-adipocyte clusters (Figure 2A) have established roles in cell development including cell-to-cell adhesion, proliferation and differentiation which have primarily been shown in synapse/neuronal cells^37–49^. It is therefore plausible that these novel genes play important yet understated roles in pre-adipocyte proliferation, commitment, and differentiation, particularly in humans. CTNNA2 was recently identified as a gene with a single nucleotide polymorphism in polyadenylation signals upregulated in mouse lines for fatness^50^. PRPTD has been identified in pre-adipocyte populations, but there is conflicting evidence on whether it contributes to or downregulates adipogenesis^51,52^. NELL1 inhibits adipogenesis in 3T3-L1 and adipose-derived stem cell lines^53^. CDH4 is among differentially expressed genes in pre-adipocytes compared to bone-resident progenitor cells and has been identified as negative regulator of UCP1^54^.

To characterize these pre-adipocyte populations further, transcription factor (TF) enrichment analysis was performed on the top DEGs (logfc >0.5) from all pre-adipocytes using ChEA3^55^ (**Supplementary Table 2**). Top ranking TFs include: 1) SATB2 which interacts with and increases transcriptional activity of pre-adipocyte marker ZNF423^56^; 2) ZNF365 which increases in expression during differentiation in pre-adipocytes^57^; and 3) CTCF a multifactorial protein required during adipogenesis^58^. Therefore, our pre-adipocyte populations express genes that can be transcriptionally regulated by known TFs during pre-adipocyte differentiation.

Cadherin-Associated Protein Alpha 2 (*CTNNA2*) was among one of the highest expressed novel pre-adipocyte genes and was ubiquitously expressed across the three pre-adipocyte populations. We validated this particular pre-adipocyte marker at the protein level using immunofluorescence staining of SVF cells collected from abdominal subcutaneous WAT (Figure 2B). We co-stained CTNNA2 with known pre-adipocyte marker CD38^36^. CTNNA2 protein was present on the majority of SVF cells but to varying degrees. CTNNA2+ cells with the highest CTNNA2 abundance were also CD38+ positive cells, confirming cells with high CTNNA2 expression are pre-adipocytes (Figure 2B).

DGE and pathway analysis between the three pre-adipocyte populations (**Supplemental Table 3**) revelated Pre-Ad 1 had an upregulation of genes encoding ribosomal proteins (*RPS6, RPL19*) indicative of greater cell proliferation^59^; whereas, Pre-Ad2 and 3 had an upregulation of gens related to synaptic and cell-to-cell signaling that was more pronounced in Pre-Ad 3. There were no proportional differences among the pre-adipocyte populations between the Older and Younger groups (Figure 2C). Mitochondrial capacity is essential for preadipocyte proliferation and differentiation^60^ and can be blunted in WAT of metabolically unhealthy individuals^61,62^. Differences in mitochondrial capacity are more pronounced in pre-adipocytes compared to *in vitro* differentiated adipocytes^63^, as putative differences between groups can be diminished following induction with an adipogenic cocktail. Using high-resolution respirometry, we observed no differences between the Older and Younger groups in basal, leak nor maximal (uncoupled) respiration (electron transport system, ETS) of cultured pre-adipocytes (Figure 2D), suggesting that mitochondrial capacity in WAT is not attenuated with aging.

#### Cell sub-clustering

We resolved adipocyte and pre-adipocyte heterogeneity in our initial clustering analyses. To resolve our remaining cell types further, we performed additional sub-clustering on our macrophage, stem cell and vascular populations. Macrophages sub-clustered into two distinct clusters (**Supplementary Figure 1A**). Macrophage_0 had markers of resident macrophages (*MRC1, PDGFC*); whereas Macrophage_1 had markers of lipid-associated macrophages (*LIPA, TREM2*, **Supplementary Figure 1B**)^64^. The increased proportion of immune cells in the Older group was due to a greater proportion of resident type macrophages (Macrophage_0) compared to the Younger group (**Supplementary Figure 1C**). There were no proportional differences in Macrophage_1 between the Older and Younger groups (**Supplementary Figure 1C**).

Stem cells sub-clustered into three stem cell populations (**Supplementary Figure 2A**). Stem_0 had upregulation of pre-adipocyte markers compared to other stem cell populations *(LSAMP, PDE4D*) indicative of committing to the adipogenic lineage. Stem_1 was defined by nascent stem cell markers (*DCN, LUM, COL1A1*) and Stem_2 had upregulation of genes associated with lipid metabolism (*PLIN4, G0S2*; **Supplementary Figure 2B**). There was a trend towards Stem_0 having a higher proportion in the Older group when expressed as a proportion of total nuclei (**Supplementary Figure 2C**). There were, however, no differences in the proportions of Stem_1 or Stem_2 between the Older and Younger groups.

Vascular cells subclustered into four cell populations (**Supplementary Figure 3A**). Vascular_0 had upregulation of lipid metabolism genes (*CIDEC, G0S2, PLIN4*) defining a fatty-acid handling endothelial phenotype as previously described^21,24^. Vascular_1 had markers of pre-ads (*DPP10, RBFOX1, PTRPD*) indicative of being a vascular originating progenitor cell^65,66^. Vascular_2 had upregulation of classical markers of endothelial cells (*PECAM1, VWF*). Vascular_3 had markers of pericytes (*PDGFRB, NOTCH3*; **Supplementary Figure 3B**). There were also no differences in proportion of the each vascular subcluster between Older and Younger groups (**Supplementary Figure 3C**).

#### Adipocyte Heterogeneity

**Figure 3:**
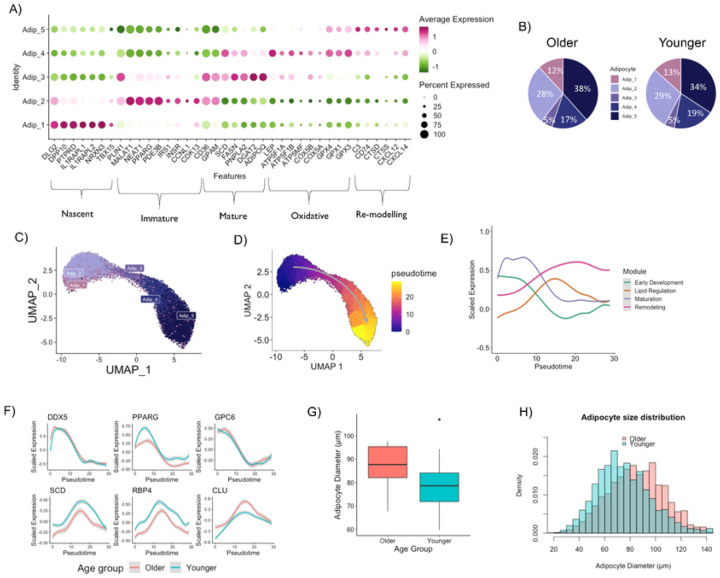
Adipocyte Heterogeneity in WAT. Gene markers defining different adipocyte populations in WAT (A). Adipocyte composition differences between older and younger participants (B). Re-clustering of the adipocyte populations (C). Pseudotime trajectory of the five adipocyte populations (D). Smoothed expression dynamics for aggregated module scores throughout the adipocyte pseudotime (E). Expression patterns of key and novel genes throughout the adipocyte pseudotime (F). Mean adipocyte diameter in older and younger groups (G). Histogram of adipocyte size distribution split by age (H).

We identified five distinct adipocyte populations and sought to identify characteristics of each cluster. We performed DGE analysis comparing each adipocyte cluster to the remaining adipocyte clusters (**Supplementary Table 4**). We characterized Adipocyte 1 as “Nascent” adipocytes due to an upregulation of genes observed in pre-adipocyte populations (*PTRPD, DPP10, NRXN3*) and lower expression of classical adipocyte markers (*ADIPOQ* and *LEP*; Figure 3A). Adipocyte 2 had an upregulation of genes associated with adipocyte growth (*PPARG, MALAT1* and *NEAT1*)^67,68^ in addition to insulin signaling genes (*INSR and IRS1*) and *PDE3B* which suppresses lipolysis^69^ and was therefore classified as “Immature” adipocytes (Figure 3A). Adipocyte 3 had upregulation of lipid synthesis genes (*GPAM, SCD, DGAT*), lipolytic genes (*PNPLA2*) and the classic adipokine *ADIPOQ* (Figure 3A). These features are characteristic of a “Mature” adipocyte carrying out routine adipocyte functions. Adipocyte 4 had upregulation of oxidative (*ATP5F1B, ATP5MF* and *COX5A*) and glutathione peroxidase (*GPX1,3–4*) genes but not typical browning or beiging markers and was therefore classified as “Oxidative”. Adipocyte 5 was defined as a “Remodeling” adipocyte due to upregulation of lysosomal proteases, cathepsins (*CTSD* and *CTSS*), complement factor (*C3*) and chemokines (*CXCL12* and *CXCL14*). There were comparable adipocyte proportions between the Older and Younger groups (Figure 3B). When divided by sex, males tended to have a higher proportion of Adipocyte 5 (Remodeling) and a lower proportion of Adipocyte 4 (Oxidative) compared to females (**Extended Figure 3A**).

#### The adipocyte continuum

We assessed whether the different adipocyte populations were end-fate phenotypes with cell-specific biological functions or rather a continuum of biological processes through time and the impact of age. We performed pseudotime analysis on the adipocyte populations after re-clustering (Figure 3C), which revealed a clear one-dimensional trajectory initiating at Adipocyte 1 (Nascent) and terminating at Adipocyte 5 (Remodeling) (Figure 3D). The pseudotime visually displayed a similar trajectory when split into Older and Younger groups (**Extended Figure 3B**). The Older group had a lower proportion of nuclei in the first pseudotemporal bin (<5 pseudotime score; 30% vs. 34%) and higher proportion of nuclei in the last psuedotemporal bin (>20 pseudotime score; 39% vs. 35%) compared to the Younger group (**Extended Figure 3C**), indicating a shift towards later stages of the adipocyte continuum in the Older group. Unsupervised hierarchical gene clustering identified four modules of genes (**Supplementary Table 5**) whose patterns changed throughout the pseudotime (Figure 3E, **Supplementary Figure 4A**), with similar patterns observed when split by age (**Extended Figure 3D, Supplementary Figure 4B-C**). The module analysis revealed that adipocyte evolution initiates with early development, followed by maturation and lipid regulation and ends with remodeling (Figure 3E, **Supplementary Figure 4**). We therefore conclude that the observed adipocyte heterogeneity (i.e. five distinct adipocyte populations) is due to different developmental stages throughout time rather than end-fate phenotypes with cell-specific functions. Importantly, the biological processes of the adipocyte continuum that explain adipocyte heterogeneity are largely retained with aging; however, the Older group has more adipocytes in the later stages of remodeling, and the Younger group has more adipocytes in the early developmental stage. While the composition and trajectory of the modules were similar between groups (**Supplementary Figure 4**), some of the key temporally-regulated genes from each module showed disparity along the pseudotime with aging (Figure 3F, **Supplementary Figure 5**). Lipid homeostasis genes (e.g. *PPARG, SRSF6, SCD, RBP4*) had elevated expression in the Younger group, whereas remodeling genes (e.g. *CLU, CFD*) were elevated in the Older group (Figure 3F, **Supplementary Figure 5**). Taken together, our data indicate that the Younger group have a greater potential to develop adipocytes with more metabolically active properties, whereas the Older group have more adipocytes actively remodeling.

#### Adipocyte Size

Differences in adipocyte size were determined by histology^70^ with an average of 158 adipocytes measured per participant. Average adipocyte diameter (μm) was not statistically different between the Older and Younger groups (Figure 3G). Distribution frequency analyses revealed that the Older group displayed a greater frequency of larger adipocytes (>90 μm) and a reduced frequency of smaller adipocytes (<70 μm) compared to the Younger group (Figure 3H, **Extended Figure 3E**).

#### Adipokines

We quantified key adipokines (adiponectin, leptin and resistin) in the plasma and conditioned media (CM) of WAT explants (**Extended Figure 3F**). There was a trend towards leptin concentrations being higher in the CM from the Younger Group compared to the Older Group (*P* = 0.09, **Extended Figure 3F**) and no differences between groups for CM adiponectin and resistin. Plasma resistin concentrations were higher in the Older Group compared to the Younger group (*P* < 0.05) with no differences plasma adiponectin and leptin **Extended Figure 3F**). Adipokine concentrations in plasma and CM had expected correlations with adipocyte size and BMI (**Supplementary Figure 6**).

### Pre-adipocyte differentiation

**Figure 4:**
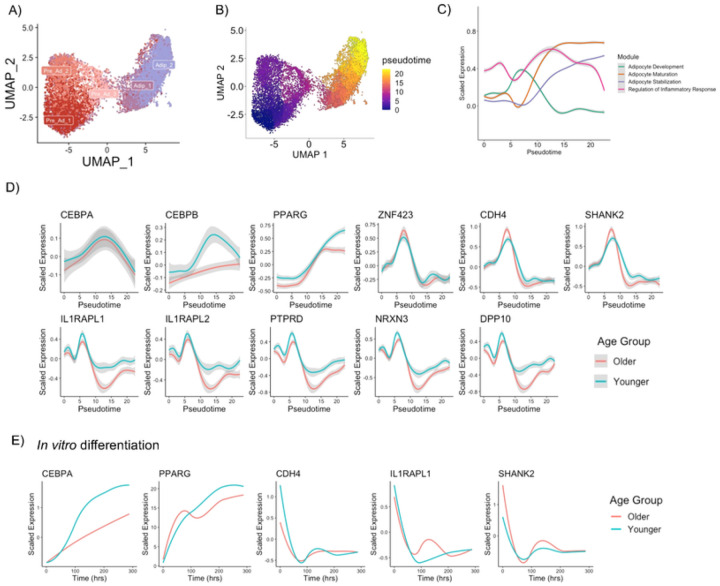
Pre-adipocyte Differentiation. Re-clustering of pre-adipocyte and early development adipocyte populations (A). Pseudotime trajectory of pre-adipocyte differentiation from pre-adipocytes to adipocytes (B). Smoothed expression dynamics for aggregated module score throughout the pre-adipocyte differentiation pseudotime (C). Expression patterns of key and novel genes throughout pre-adipocyte differentiation pseudotime (D). Expression patterns of key and novel genes through pre-adipocyte differentiation *in vitro* from Older (N = 4) and Younger (N= 3) individuals (E).

We next sought to track pre-adipocyte differentiation *in vivo* using a pseudotime trajectory. We re-clustered the pre-adipocyte populations with Adipocytes 1 (Nascent) and 2 (Immature) which are in the earlier stages of adipocyte development (Figure 4A). The pseudotime trajectory initiates in Pre-ad 1, branches off to Pre-ad 2 or 3, with Pre-ad 3 then converting towards Adipocyte 1 and Adipocyte 2 (Figure 4B). Similar pseudotime trajectories and similar proportions of nuclei in each pseudotemporal bin were observed between Older and Younger groups (**Extended Figure 4A-B**). Unsupervised hierarchical gene clustering identified four modules of genes (**Supplementary Table 6**) whose patterns changed throughout the pre-adipocyte differentiation pseudotime (Figure 4C, **Supplementary Figure 7A**), with similar patterns observed when split by age (**Extended Figure 4C, Supplementary Figure 7B-C**). The module analysis revelated that pre-adipocyte differentiation initiates with adipocyte development, transitions with adipocyte maturation and stabilization and has a fluctuation of inflammatory responses throughout (Figure 4C, **Supplementary Figure 7**). To validate our pre-adipocyte differentiation pseudotime, we plotted known markers of pre-adipocyte differentiation (*CEPBA, CEBPB & PPARG*; Figure 4D). As expected, early regulators of pre-adipocyte differentiation *CEBPA* and *CEBPB* increased during the early phases of the pseudotime (Figure 4D), while *PPARG* continues to increase throughout the pseudotime (Figure 4D). Both *CEBPB* and *PPARG* had a blunted response in the Older group (Figure 4D). Key genes from Module 1–3 showed little disparity with aging when transitioning along the pseudotime (**Supplementary Figure 8**). Due to module 4 expressing genes that align with commitment of pre-adipocytes to the adipogenic lineage, we mapped these novel markers along the pseudotime (Figure 4D). Known committed pre-adipocyte marker *ZNF423* was used as reference. Several of these genesdisplayed a similar pattern to *ZNF423* exhibiting a rapid increase at pseudotime score 7–8 corresponding to pre-adipocyte commitment (Figure 4D). Interestingly, the expression of the genes declined as the pseudotime transitioned to a score of 10–15 which corresponds to a Nascent adipocyte being formed and where a rapid increase in *PPARG* is observed (Figure 4D). This decline was dampened in the Younger group which retained elevated expression of several of these genes (*IL1RAPL1, IL1RAPL2, PTPRD, NRXN3 & DPP10*) as the pseudotime progressed (Figure 4D). We propose that previously assigned neuron/synapse genes play an essential role in forming committed pre-adipocytes and early adipocyte development and the elevated expression in the Younger group indicates a more poised state for differentiation during early development.

To further validate a subset of these novel pre-adipocyte markers, we tracked the expression levels during differentiation *in vitro*. Cultured pre-adipocytes derived from digested human subcutaneous abdominal WAT were differentiated using an established adipogenic cocktail^71^ and cells were harvested for RT-qPCR at time-points Day 0, Day 0 + 4hrs, Day 2, Day 4, Day 9 and Day 12 (**Extended Figure 4D**). To match the *in vivo* pseudotime trajectory, Days were converted to hours and gene expression patterns were modelled throughout the differentiation period. As anticipated, known adipogenesis markers CEBPA and *PPARG* increased with differentiation (Figure 4E). Novel pre-adipocyte markers from Module 4, *CDH4, SHANK2* and *IL1RAPL1* had their highest expression levels at time point 0 before the differentiation cocktail was added and when they would still be classified as pre-adipocytes. This aligns with a pseudotime score 7–8 corresponding to pre-adipocyte commitment *in vivo. CDH4, SHANK2* and *IL1RAPL1* expression rapidly decreased upon addition of the differentiation cocktail coinciding with the cells rapidly transitioning to adipocytes, with no discernable differences between the Older and Younger groups. Our data confirm that these are pre-adipocyte markers are temporally regulated during pre-adipocyte differentiation. We posit, however, that nuances in gene expression levels between groups are better detected using an *in vivo* pseudotime approach rather than artificial induction *in vitro*.

### Cell-Type transcriptional differences between Older and Younger groups

#### Random Forest Classification

**Figure 5:**
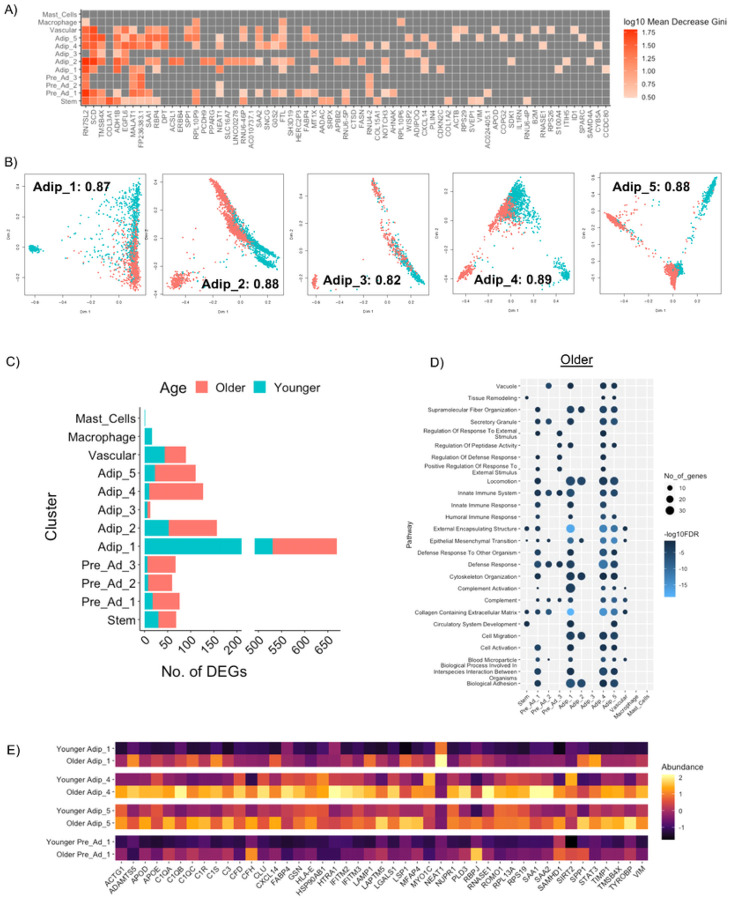
Transcriptional differences between old and young cell populations. Top genes contributing to random forest classification for Older and Younger groups based on each cell cluster (A). Multi-dimensional scaling plots using top genes that contribute to random forest classification accuracy for each adipocyte cluster showing differences between the Older and Younger group. The number included is the accuracy score for that clusters (B). The number of DEGs between Older and Younger for each cluster (C). Top pathways upregulated in the Older group for each cluster (D). Heatmap of genes contributing to immune response and complement activation highlight differences between the Older and Younger Pre_Ad_1, Adip_1, Adip_4 and Adip_5 (E).

Random forest classification^72^ was performed on each cluster to determine the genes most important in classifying an Older and Younger cell for that specific cluster (**Supplementary Table 7**). The top genes based on a Mean Decrease Gini above 3 are shown in Figure 5A. Out of the 12 separate clusters, the five Adipocyte clusters, Pre-adipocyte 1, Vascular cells and Stem cells showed the highest accuracy (> 0.8) of random forest classification (Figure 5B, **Extended Figure 5A**), meaning that cells from Older and Younger individuals can be classified more accurately if selected at random from these clusters. Among the top genes contributing to this high accuracy that were also upregulated in Older individuals were *DPT* and *CXCL14*. DPT regulates extracellular matrix (ECM) formation and is upregulated in WAT in obesity-induced T2D^73^. Treatment of isolated adipocytes with DPT increases gene expression of ECM regulators (*COL6A3, ELN, MMP9, TNMD*) and inflammatory markers (*IL6, IL8, TNF*)^73^. CXCL14 is notable for its chemotactic properties^74^ but has recently been identified as a mediator of macrophage communication in brown adipose tissue^75^, as well as a regulator of insulin-mediated glucose uptake in 3T3-L1 adipocytes^76^. Together, these genes indicate an enhanced immune response in cells from the Older group. Among the top genes contributing to high accuracy that were also upregulated in Younger individuals were genes related to lipid metabolism (*SCD, FASN, G0S2, ACSL1* and *ADH1B*) and adipogenesis (*PPARG*), indicative of active routine regulatory processes typical in WAT. Macrophages and Mast Cells had the lowest accuracy scores suggesting lower transcriptional diversity between Younger and Older groups for these cell types (**Extended Figure 5A**).

#### Differential gene expression

We performed DGE analysis comparing Older and Younger groups for each cell cluster (**Supplementary Table 8**). The Older group had the highest number of upregulated genes among the cell clusters (Figure 5C) except for Adipocyte 1 (Nascent adipocyte). Pathway analysis revealed the main pathways upregulated in the Older group were related to innate immune response and complement activation and were observed to the greatest extent in in Pre-Ad 1, Adipocyte 1 (Nascent adipocyte), Adipocyte 4 (Oxidative adipocyte) and Adipocyte 5 (Remodeling adipocyte) (Figure 5D). Genes contributing to immune response and complement activation pathways are shown in Figure 5E. In addition to *bona fide* immune cells, adipocytes themselves are now recognized as key innate immune cells within adipose tissue^77^. The data suggests that in the Older group, these pre-adipocyte and adipocyte populations are initiating an innate immune response via alternative complement pathway activation. As part of the innate immune response, these cells undergo remodeling of ECM (*ADAMTS5, VIM, HTRA1, MFAP4, SPP1, TIMP1*), phospholipid membrane (*PLD3*), lipids (*APOD, APOE*,) actin cytoskeleton (*ACTG1, GSN, LSP1, MYO1C*) and carbohydrate binding structure (*LGALS1, RNASE1*) that is in part mediated by lysosomal activity (*LSAM1, LAPTMS*) and ROS regulation (*ROMO1*). In the Younger group, the main pathways upregulated were related to neurogenesis and synapse organization in Adipocyte 1 (**Extended Figure 5B**). These genes have previously been attributed to the development of neurons; however, given their upregulation in Adipocyte 1 (Nascent) and in agreement with our pre-adipocyte differentiation pseudotime, we again reason that these genes are early regulators of pre-adipocyte differentiation.

### Senescence

**Figure 6:**
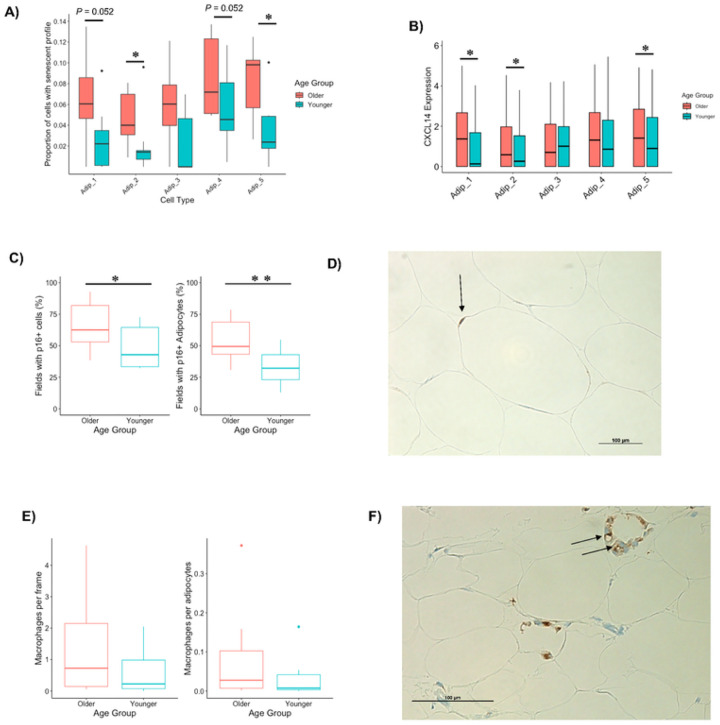
Markers of senescence in aging WAT. The proportion of nuclei with a high senescence profile (top 10%) in adipocyte populations between Older and Younger participants (A). CXCL14 expression (B). The percentage of 20x magnification fields that had cells and adipocytes with p16 staining between Older and Younger participants (C). Representative image of p16 cell staining from an Older individual with p16 in and adipocyte highlighted (D). The number of macrophages per frame and per adipocytes (E). Representative image of macrophage crown like structure in the the Older group (F). *, *P* < 0.05; **, *P* < 0.01.

Cellular senescence is caused by accumulation of DNA damage^78^ and other cellular stressors^79–81^, leading to enlarged cells that are resistant to regulated cell death^82^ and secrete chemokines, cytokines, growth factors, and matrix metalloproteinases^83^ that can negatively impact the function of other cells in the microenvironment^11,84^. Senescence typically occurs in proliferating cell types but has also been found in terminally differentiated cells^85,86^ including adipocytes^9,87^. Senescent cells activate innate immunity to assist in the removal of nearby dying cells^10,88^. Given that the Older group has larger (ie, hypertrophic) adipocytes, upregulated innate immunity specifically in adipocyte populations and increased proportions of adipocytes in the end-stage of the adipocyte pseudotime, we reasoned that the Older group would have increased levels of senescence, particularly among their adipocytes. p16 (CDKN2A) and p21 (CDKN1A) are canonical markers of senescence but are unreliable in detecting senescence at the transcriptional level due to very low expression. To measure senescence profiles in our snRNA-Seq data set, we performed GSEA on each nucleus using the recently published SenMayo gene set which identifies the transcriptional profile of senescent cells with a combination of 125 genes^89^ (**Extended Figure 6A**). In line with Saul *et al*.^89^, we determined that the top 10% of cells could be classified as those with a senescent profile. The Older group had a significantly greater proportion of cells with a senescent profile in Adipocyte clusters 2, 5 and Pre-Adipocyte 1 and a trend towards a greater proportion of cells with a senescent profile in Adipocyte clusters 1 and 4 (Figure 6A, **Extended Figure 6B**). It was notable from our data set, that the only chemokine or cytokine upregulated in clusters that have an increased proportion of cells with a senescent profile was *CXCL14* (Figure 6B). This gene was also included as high importance in distinguishing between Older and Younger individuals in our prior random forest classification (Figure 5A).

To confirm Older individuals had a greater proportion of cells with a senescent profile, we performed immunohistochemistry staining for the classic senescent marker p16. Abundance of p16+ cells was quantified as the percentage of 1) total fields (20x magnification) analyzed containing p16+ cells^9^ and 2) p16+ adipocytes. The Older group had significantly more fields containing p16+ cells than the Younger group (Figure 6C–D). The percentage of fields containing p16+ adipocytes was also greater among Older individuals (Figure 6C), suggesting the increased senescence profile in the Older group is driven by adipocytes and aligns with the snRNA-seq data (Figure 6A–B).

Given that CXCL14 may mediate macrophage infiltration^90^ and that our snRNA-seq data show a greater number of resident macrophages in the Older group, we quantified macrophage content via immunohistochemistry using a pan-macrophage marker CD68^91^. The numbers of macrophages quantified with CD68 per frame and per adipocyte were not statistically significant between the Older and Younger groups (Figure 6E). Three participants had crown-like structures of macrophages (Figure 6F), which form around dying or damaged adipocytes^92^. All of these individuals were male and two of them were from the Older group. Obesity is associated with macrophage infiltration^91^. In agreement, we noted a significant and positive correlation between a marker of abdominal obesity, waist-hip-ratio (WHR), and the number of macrophages per adipocytes (**Extended Figure 6C**). WHR was greater in the Older group (Table 1), and this was driven specifically by Older males (**Extended Figure 6D**). We therefore reason that macrophage infiltration occurs in Older males with higher WHR that can lead to crown-like structures developing around damaged adipocytes, perhaps as a means to maintain healthy WAT.

## Discussion

We present a single nuclei atlas of aging human abdominal subcutaneous WAT in the largest prospective cohort study to date using cutting-edge full-length transcriptional profiling. We highlight new markers of pre-adipocyte populations that are temporally regulated during pre-adipocyte differentiation. We show that different adipocyte populations do not become distinct end-fate phenotypes with specific functions, but rather each adipocyte transitions through a developmental state along a continuum.

In the context of healthy aging, the main findings were that specific adipocyte populations in the Older group have an increased expression of genes related to innate immune response and an increased proportion of adipocytes with a senescent profile. Phenotypically, the Older group had a shift towards larger adipocytes which have been associated with hypoxia, oxidative stress and inflammation^93,94^. Resident-like (M2) macrophages are fundamental in providing an initial defense mechanism during the innate immune response^95^ and in agreement, the Older Group had increased proportions of resident-like macrophages. These differences exist despite the Older group having comparable whole-body and WAT metabolic health to the Younger group. We therefore propose that aging WAT endures very low-grade inflammation insults that are managed through their innate immunity foundation to preserve the metabolic health of the adipose tissue. CXCL14 was the only chemokine or cytokine upregulated in these specific adipocyte populations in the Older group. The role of CXCL14 in WAT has been confounded by a lack of consensus identifying and validating its receptor; however, it was recently proposed that CXCL14 synergizes with low concentrations of CXCL13 and CCL19/CCL21 during *in vitro* chemotaxis with immune cells expressing receptors CXCR5 and CCR7^96^. CXCL14 is known to recruit M2 macrophages^75^ and its adipose tissue expression and circulating concentrations are reduced with metabolic abnormalities such as obesity, PCOS and T2D^90,97^, suggesting it may be a therapeutic candidate that mediates low-grade inflammation to maintain metabolic health of the tissue. Future research is needed to examine if the innate immunity foundation that preserves metabolic health in WAT is impaired in an unhealthy human aging model that is paired with metabolic and cardiovascular disease, as well as impairments in physical and physiological function.

## Methods

### Experimental model and subject details

Younger (≤ 30 years old) and Older (≥ 65 years old) individuals were recruited to the Translational Research Institute at AdventHealth to participate in the study. Full inclusion/exclusion criteria can be found in Supplementary information 1. Briefly, all participants were free from metabolic and infectious disease, were not taking medication related to diabetes or inflammation and had not had major surgery within the last 4 weeks. Five participants were taking 1 or more medications to treat the following conditions; hypertension, hyperlipidemia, urinary retention, thyroid, anxiety and osteoporosis. All participants were weight-stable for at least 3 months prior to the assessments and adipose biopsy. The study was approved by AdventHealth Institutional Review Board and carried out in accordance with the Declaration of Helsinki. Participants provided written informed consent to partake in the study.

Abdominal subcutaneous WAT biopsies were performed following an overnight fast using the tumescent lidocaine approach with a Mercedes aspiration cannula^98^. Following removal of excess blood and connective tissue, the sample was cleaned with PBS. A portion (~100mg) was immediately snap frozen for subsequent nuclei isolation for snRNA-seq. A portion (~20mg) was fixed in 10% Formalin for 24 hours and stored in 70% EtOH for subsequent histological analyses. A portion (~100mg) was used as an explant to collect conditioned media. The remaining tissue was stored in Medium 199 containing 25mM HEPES at room temperature prior to digestion of adipose derived progenitor cells and *in vitro* experiments.

### Nuclei isolation from whole white adipose tissue

Nuclei was isolated from frozen WAT as previously detailed^26^. WAT was pulverized under linked nitrogen before being homogenized in 2mL of homogenization buffer (5mM MgCl2, 25mM Tris Buffer pH 8.0, 25mM KCL, 250mM sucrose, 1μm DDT, 1 x protease inhibitor, 0.2 U/μL SUPERase. In RNase Inhibitor (Thermofisher Scientific) in nuclease-free water) with a glasscol homogenizer. Following addition of Triton-X100 (0.1% v/v) the homogenate was incubated on ice with regular vortexing. Samples were then filtered through a 100μm and 40μm strainer (BD Falcon), centrifuged at 2,700g for 10 min at 4°C, resuspended in homogenization buffer and recentrifuged again at 2,700g for 10 min at 4°C. The pellet was then re-suspended in 1mL nuclei isolation medium (5mM MgCl2, 25mM Tris Buffer pH 8.0, 25mM KCL, 1 mM EDTA, 0.2 U/μL Ribolock RNAase inhibitor, 1% BSA in nuclease-free water) before being centrifuged at 2,700g for 10 min at 4°C. The sample was re-suspended in 500μL nuclei isolation medium before being filtered 10 x with a 25g syringe. Nuclei was stained with Hoechst 33342 (ReadyProbes Cell Viability Imaging Kit, Thermofisher Scientific) and counted with a countess II automated cell counter (Thermofisher Scientific).

### Isolation of human adipose-derived progenitor cells

Abdominal subcutaneous WAT was minced and digested with collagenase (type 1, 1 mg/ml, 3% BSA in HBSS) (Worthington Type I Collagenase cat# S004196) using 1 g/ 2ml ratio, for 30 – 60 min at 37°C with shaking (100rpm), as described previously^71^. The mixture was passed through a 250 μm mesh, rinsed with HBSS, centrifuged at 200*g* for 5 minutes, and floating mature adipocytes were removed. Progenitor cells were span at 500*g* for 5 minutes and cell pellet was treated with erythrocyte lysis buffer (RBC lysis buffer, cat# 420301). Cells were re-pelleted with centrifugation, resuspended with alpha Dulbecco’s modified Eagle’s Medium (αMEM) + GlutaMax (Gibco, cat# 32561–037) supplemented with 10% fetal bovine serum (FBS), 100U/ml penicillin and 100μg/ml streptomycin, filtered through 100μm cell strainer, and then plated and culture in a humid atmosphere with 5% CO_2_. After 4 hrs, non-adherent cells were removed, and media was replaced. Adherent cells were allowed to reach 80% confluence with medium changed every other day and then either subculture or frozen (media supplemented with 10% DMSO and 16% FBS) for cryopreservation.

### Single-nuclei RNA-Seq

Single-nuclei suspension (40K/mL) was aliquoted into 8 wells of a 384-well source plate (Takara Bio USA, San Jose, CA) and dispensed using an iCELL8 MultiSample NanoDispenser (Takara Bio USA) onto an iCELL8^®^ 350v Chip (Takara Bio USA. Following dispense, the chip nanowells were imaged using the iCELL8 Imaging Station to identify nanowells containing a single nucleus, with only these nanowells being subjected for downstream dispenses. After imaging, the chip was subjected to freeze-thaw to lyse the nuclei, followed by a 3 min incubation at 72°C to denature the RNA. Selected nanowells were subjected to first-strand cDNA synthesis initiated by oligo dT primer (SMART-Seq iCELL8 CDS), followed by template switching with template switching oligo (SMART-Seq iCELL8 oligonucleotide) for 2^nd^ strand cDNA synthesis, before unbiased amplification of full-length cDNA. Tagment DNA enzyme 1 (TDE1, Illumina, San Diego, CA) was used to tagment full-length cDNA before amplification with forward (i5) and reverse (i7) indexing primers. Each single nucleus was indexed by a unique combination of 1 of 72 forward and 1 of 72 reverse indexing primers allowing for downstream identification. Collected cDNA was purified twice using a 1:1 proportion of AMPure XP beads (Beckman Coulter, Brea, CA). cDNA was further amplified according to manufacturer’s instructions and purified again at a 1:1 proportion of AMPure XP beads. The resultant cDNA library was assessed for concentration by fluorometer (Qubit, Thermofisher Scientific) and quality by electrophoresis (Agilent Bioanalyzer high sensitivity DNA chips). Libraries were sequenced with Illumina HiSeq 4000 at an average sequencing depth of 270 M per library. This equated to an average 118,568 barcoded reads per nuclei.

#### Bioinformatic analyses

Initial analyses of the single nuclei libraries were performed using CogentAP^™^ Analysis Pipeline (Takara Bio, USA). GRCh38 was used as the genome reference. Cell and gene filtering was performed in R package scran and scuttle^99^. Nuclei were initially filtered if they had; < 500 genes, > 20% mitochondrial reads or if cell complexity was < 0.65. Following initial filtering, outliers were removed based on 3 median absolute deviations of log total counts. A minimum threshold of 0.1 was applied to filter out low expressed genes. All 20 samples were integrated and normalized in Seurat with SCTransform using 5000 highly variable protein coding genes^100,101^. Dimensional reduction to obtain integration anchors was achieved with reciprocal PCA. Once integrated, significant principal components were used to perform unsupervised K-nearest neighbor (KNN) graph-based clustering. Visualization was achieved with uniform manifold approximation and projection (UMAP). Differential gene expression analysis for cell clusters was performed using a Wilcoxon rank sum test with Seurat’s “FindMarkers” function with a FDR cut off of < 0.05, log_2_ FC > 0.25 or < −0.25 and expressed in >25% of nuclei in that cluster. Differential gene expression analysis between the Older and Younger group was performed using a Wilcoxon rank sum test with Seurat’s “FindMarkers” function with a FDR cut off of < 0.05, log_2_ FC > 0.1 or < −0.1 and expressed in >10% of nuclei in that cluster. A hypergeometric test was used to assess over-representation of upregulated genes (log_2_ FC < 0.25) in the Older and Younger group for each cell type using R package HypeR^102^ querying datasets of; gene ontology, KEGG, Reactome and Hallmark^103–107^. Significance was set at an FDR of 0.05. Genes detected in each cluster was used as a background reference. Senescent enrichment score was generated using R Package Ucell and function enrichIt()^108^, with the SenMayo list of genes used as the gene set^89^.

#### Pseudotime trajectory

Monocle 3 was used for trajectory analysis^109–113^. After analyzing the dynamic biological changes of each nucleus, an individual position of every single nucleus is plotted in a learned trajectory. Based on the clustered annotation and marker genes, we identified the root of the given trajectory and ordered nuclei along the pseudotime according to their developmental progress. For adipocyte lifecycle we identified this root as Adipocyte 1 and for the adipocyte differentiation we identified the roots as Pre-Ad 1 and 2. The nuclei were split into 5 pseudotemporal bins based on their pseudotime score (<5, 5–10, 10–15, 15–20, >20). Temporally regulated genes were identified by performing DGE (logfc > 0.8 & FDR < 0.05) for each bin against the first and last pseudotemporal bin as previously described^114,115^. Unsupervised hierarchical gene clustering was performed by K-means clustering using R package complex heatmap^116^ to identify modules of genes that change throughout the pseudotime. A hypergeometric test was used to assess over-representation of genes within a module using R package HypeR^102^ querying data set of gene ontology^106,107^. Significance was set at an FDR of 0.05. Aggregated gene expression for each module was calculated using the AddModuleScore() function in Seurat, allowing aggregated module gene expression to be plotted throughout the pseudotime.

#### Pairwise correlation analysis

We compared gene expression profiles between the current data set and previously published data sets. Initially samples were integrated together using Seurat as described above. Barcodes of cells/nuclei comprising the main cell types for each analysis were used to define the main cell populations. Correlation analyses were performed among these different cell populations.

#### Random Forest

Random Forest classification was used to rank genes on their importance in distinguishing between the Older and Younger group for each cell type^72^. The cell types were randomly split into a training (80%) and testing set (20%) to complete and evaluate modelling. Random Forest modelling was computed on each cell type to predict nuclei that originated from the Older and Younger groups. Accuracy scores for each cell type was computed and mean decrease gini was used to measure variable importance in the classification. Multi-dimensional scaling plots were generated using the top genes that contribute to the random forest classification accuracy for each cell-type.

### Pre-adipocyte differentiation *in vitro*

Pre-Adipocytes derived from Younger (N = 3) and Older (N = 4) individuals were thawed from −80°C at a low passage, seeded and differentiated as previously described^71^. Briefly, 2-day post-confluency cells were induced to differentiate in DMEM/Ham’s F12 (1:1) containing isobutylmethylxanthine (0.5 mm), dexamethasone (100 nm), insulin (100 nm), T3 (2 nm), and transferrin (10 μg/liter) for 6 days and then maintained in DMEM/F12 containing only insulin (10 nm) and dexamethasone (10 nm) until day 12. Cells were harvested at; Day 0, 4 hrs, Day 2, Day 4, Day 9 and Day 12. RNA was extracted using Rneasy kit (Qiagen) and cDNA was generated using reverse transcriptase. Target Genes and housekeeping gene (PPIA) were measured by RT-qPCR using a ViiA7 sequence detection system (Life technologies) and SyberGreen technology suitable for relative gene expression quantification using the following parameters: one cycle of 95°C for 10 minutes, followed by 40 cycles at 95°C for 15 seconds and 60°C for 1 minute.

### Immunofluorescence staining of stromovascular fraction (SVF)

A cover slip was added in each well of a 24-well tissue culture plate. SVF were seed at a density of 140K/well and kept in culture overnight in αMEM supplemented with 10% FBS. Non-adherent cells were washed with PBS. The remaining adherent cells (ADSCs, endothelial cells and preadipocytes) were fixed with 4% paraformaldehyde for 30min. The plate was washed 3 times with PBS and kept at 4°C until IF staining was performed. Cells were incubated with 0.1M glycine in PBS for 15 minutes before being permeabilized for 5 min with PBS containing 1% NP40 and 0.05% saponin. Cells were blocked for 30 minutes using blocking solution (5% NGS in 0.1M PB). Cells were stained with primary antibodies targeting CTNNA2 (LSBio, #LS-C669704–50, dilution 1:50) and CD38 (abcam, #ab23518, dilution 1:200) overnight at 4°C, washed 6 times for 5 min with PBS and then incubated with secondary alexa fluor antibodies (Thermofisher; GARIgG 488 (#A32731), GAMIgG 633 (#A21052) for 45 min in the dark at room temperature. Following another 6 × 5 min PBS washes, cover slips were mounted onto slides with ProLong Gold Antifade mountant containing DAPI (Thermofisher, #P36941). Images were captured using a Nikon eclipse Ti microscope (Nikon Technologies, California) at 40x magnification.

### Adipose Tissue Histology

Adipose tissue stored in 70% EtOH was embedded in paraffin and sectioned at 5 μm. Slides were stained with H&E to determine adipocyte sizing. Immunohistochemical detection of CD68 (Atlas Antibodies, AMAb90873) for macrophages and p16 (Enzo Life Science, ENZ-ABS664) with the avidin-biotin peroxidase method. Images were captured using a Nikon eclipse Ti microscope (Nikon Technologies, California) at 20x magnification.

Adipocyte size was quantified using ImageJ software. An average of 158 adipocytes were measured per participant from 6 frames. Each adipocyte was manually outlined to create an ROI, then the area was calculated by the ImageJ software. Adipocytes that were below 200μm^2^ and above 16,000μm^2^ were removed as they typically represent artefacts from processing^70^. Abundance of p16+ cells were quantified as the percentage of total fields (20x magnification) analyzed containing p16+ cells^9^ and p16+ adipocytes over an average of 26 frames per participant. Head-and-neck cancer tissue was used as a positive control for the presence of p16. The presence of CD68-positive cells was counted per frame with an average of 18 frames per participant and adjusted for the number of adipocytes per frame for each participant. Presence of crown-like structures for each frame was also noted. Tonsil tissue was used as a positive control for the presence of CD68.

### Measurement of mitochondrial capacity (oxygen consumption rates)

Oxygen consumption (mitochondrial capacity) rates were performed via high resolution respirometry using the Oxygraph-2K (Oroboros Instruments, Innsbruck, Austria). Cultured pre-adipocytes (passages 3 to 6) derived from Younger (N = 5) and Older (N = 5) male individuals were plated in in 6 well/plate with 62,500 cells/well in αMEM supplemented with 10% FBS for 48 hrs to reach 80% confluency. Cells were detached from plates using StemPro Accutase (Gibco, cat#A11105–01)), centrifuged and resuspend in DMEM (Gibco, cat#11885–084)) medium. 3 wells of cells were used per chamber of the Oxygraph. Basal respiration was performed in intact cells. Addition of 1 μg/mL of oligomycin (Sigma-Aldrich, cat # 04876–5MG) and 0.2 mM of mitochondrial uncoupler (FCCP, Sigma-Aldrich, cat# C2920) allowed for measurements of Leak and maximal capacity of the electron transport system (ETS), respectively. Oxygen flux was normalized to cell number.

### Human adipose tissue conditioned media

Adipose tissue was incubated in M199 media (Gibco, cat# 11043–023) containing 25 mM Hepes using a 20:1 ratio (μl:mg) in a 50 mL falcon tube and cultured in a water bath shaker (100 rpm) at 37°C for 3 hrs. After that period, tissue was collected by using a 100 μm cell strainer and conditioned media was centrifuged at 500 g for 5 min, then aliquoted and frozen at −80°C. Adipokine; leptin, adiponectin and resistin were measured by ELISA using reagents from the R&D systems, Inc. (cat# DLP00, cat# DRP300 and cat# DRSN00).

#### Blood metabolites

Fasting blood samples were collected for measurements of comprehensive metabolic panel, HbA1c (%), insulin, FFA and CRP and analyzed in the clinical chemistry laboratory at AdventHealth using standard assays. Adipose tissue insulin resistance index (ADIPO-IR) was calculated by multiplying plasma FFA (mmol/L) by serum insulin (pmol/L)^27,28^.

### Statistical testing

For comparisons of clinical and phenotypical data between the Older and Younger group, an unpaired t-test was used to detect differences in normally distributed data and Mann-Whitney U test was used for non-normally distributed data. A two-way ANOVA was used when comparing variables for age and sex with post-hoc Tukey HSD.

## Figures and Tables

**Table 1:** 

Clinical Variable	Older	Younger	*P* value
Age (years)	74 ± 7	26 ± 3	***1.19E*** ^***−10***^
Sex (F/M)	5/5	5/5	
Race (WH/BL/AI/UNK)	(8/1/0/1)	(7/2/1/0)	
Ethnicity (NH/H/UNK)	(5/1/4)	(2/5/3)	
BMI (kg/m^2^)	29.38 ± 4.78	28.31 ± 4.80	0.623
Waist (cm)	102.69 ± 13.39	90.81 ± 8.07	***0.0272***
Waist-to-hip Ratio	0.96 ± 0.11	0.86 ± 0.08	***0.0252***
SBP (mmHg)	136 ± 13	122 ± 9	***0.0256***
DBP (mmHg)	78 ± 8	72 ± 7	0.109
Glucose (mg/dL)	97.50 ± 15.71	89.39 ± 7.23	0.151
Insulin (μIU/mL)	13.52 ± 7.36	11.34 ± 6.20	0.497
HbAlc (%)	5.63 ± 0.27	5.26 ± 0.54	0.0694
Cholesterol (mg/dL)	187.90 ± 43.51	151.20 ± 26.37	***0.0349***
LDL (mg/dL)	111.30 ± 42.58	80.60 ± 26.68	0.0692
VLDL (mg/dL)	21.10 ± 8.35	13.20 ± 6.32	***0.0406***
HDL (mg/dL)	55.50 ± 11.32	57.40 ± 12.20	0.722
Non-HDL Cholesterol (mg/dL)	132.40 ± 44.76	93.80 ± 24.53	***0.0279***
Triglycerides (mg/dL)	104.90 ± 41.95	66.10 ± 31.80	***0.0316***
FFA (mmol/L)	0.26 ± 0.14	0.34 ± 0.20	0.374
ADIPO-IR	0.56 ± 0.33	0.69 ± 0.52	0.522
TSH (μIU/mL)	2.59 ± 1.19	1.52 ± 0.78	***0.0279***
ALT (units/L)	20.00 ± 3.13	26.90 ± 28.47	0.361
AST (units/L)	26.00 ± 2.16	25.70 ± 19.56	***0.0278***
CRP (mg/L)	2.43 ± 1.61	2.68 ± 2.82	1

Abbreviations: ADIPO-IR, Adipose Tissue Insulin Resistance Index; AI, American Indian; ALT, Alanine Transaminase; AST, Aspartate Aminotransferase; BL, Black; BMI, Body Mass Index; CRP, C-Reactive Protein; DBP, Diastolic Blood Pressure; F, Female; FFA, Free-fatty Acid; H, Hispanic; HbA1c, Hemoglobin A1C; HDL, High-Density Lipoprotein; LDL, Low-Density Lipoprotein; M, Male; NH, Non-Hispanic; SBP, Systolic Blood Pressure; TSH, Thyroid Stimulating Hormone; WH, White; UNK, unknown;

## Data Availability

snRNA-Seq data generated during this study have been deposited under GSE235529.
